# Coronaviruses remodel the mature human tRNAome to modulate infection

**DOI:** 10.1080/21505594.2025.2580129

**Published:** 2025-10-28

**Authors:** Yining Wang, Xin Wang, Amine Avan, Pengfei Li, Xumin Ou, Qiuwei Pan

**Affiliations:** a Department of Gastroenterology and Hepatology, Erasmus MC-University Medical Center, Rotterdam, The Netherlands; b Engineering Research Center of Southwest Animal Disease Prevention and Control Technology, Ministry of Education of the People’s Republic of China, Chengdu, Sichuan, China; c Key Laboratory of Animal Disease and Human Health of Sichuan Province, Sichuan Agricultural University, Chengdu, Sichuan, China

**Keywords:** Coronaviruses, tRNA, amino acid, infection

## Abstract

Current research on virus-host interactions primarily focuses on the transcription and translation of viral and host genes. However, there is a major knowledge gap between transcription and translation, known as translational decoding mediated by mature transfer RNAs (tRNAs) charged with amino acids. Codon usage analysis of seven human coronaviruses indicates that they are highly dissimilar from the human host. Quantification of the human tRNAome, consisting of 57 species, demonstrated that infections with these coronaviruses robustly upregulate the global tRNAome landscapes in host cells. Deprivation of individual amino acids or knockdown of TRNT1, the enzyme adding 3’-ACC terminal for tRNA aminoacylation, inhibited coronavirus infection. Integrative analysis of codon usage and the tRNAome landscape identified a prominent role of tRNA-Asn-AUU in translational decoding of different human coronaviruses. Deprivation of asparagine (Asn) or knockdown of Asparaginyl-tRNA Synthetase 1, an enzyme that charges the Asn amino acid onto tRNA-Asn acceptors, including tRNA-Asn-AUU, profoundly inhibited coronavirus 229E infection, but to a much lesser extent for NL63 and SARS-CoV-2. Collectively, we demonstrated that human coronaviruses are capable of remodeling the host mature tRNAome to facilitate infection. However, the regulatory patterns and sensitivities to interference, particularly at the single amino acid or tRNA levels, vary among different coronavirus species. These findings provide a new perspective for understanding virus-host interactions.

## Introduction

Coronaviruses are a large family of single-stranded positive-sense RNA viruses that circulate in various natural hosts. Bats and birds are the main natural reservoirs of many coronaviruses. To date, seven types of coronaviruses have been known to infect humans-four seasonal (OC43, 229E, NL63, and HKU1) and three highly pathogenic (SARS-CoV, SARS-CoV-2, and MERS-CoV) coronaviruses [1]. The coronavirus species circulating in animal reservoirs pose major public health threats, because they can jump and adapt to humans to cause epidemics [2].

Various mechanisms regulate the success of cross-species transmission and the subsequent adaptation to new hosts. In turn, the host has powerful defense mechanisms against viral invasion [3]. The intricate process of virus-host interactions determines host adaptation, infection course, pathogenesis, and eventual outcomes [4]. Current research on understanding the early events of cross-species transmission primarily focuses on the viral genomic mutations related to receptor binding and viral entry. The understanding of virus-host interactions post-entry mainly focuses on transcription and translation of viral and host genes [4]. However, there is a major knowledge gap between transcription and translation, known as translational decoding, operated by transfer RNAs (tRNAs) charged with the corresponding amino acids [5].

Cellular tRNAs are transcribed from genomic DNA and are typically 70–90 base pairs in length often with multiple genomic copies of the same tRNA. The human tRNAome is encoded by more than 600 tRNA loci [6]. Following transcription, tRNAs are subjected to post-transcriptional modification, and a common CCA ribonucleotide sequence is added to the 3’ end to obtain the mature form. The CCA tail of mature tRNA is the site where an amino acid is charged, which is catalyzed by aminoacyl-tRNA synthetases, providing substrates for protein synthesis [5]. Theoretically, the mature tRNAome comprising 61 types of aminoacyl-tRNAs would be required for decoding the 61 triplet codons that specify 20 amino acids. However, the minimum number of tRNA species required for decoding in real life is usually less than the theoretically calculated number because of the wobble decoding rule [7].

All viruses depend solely on the host translation machinery to produce viral proteins. Both viral and host genes are decoded in ribosomes to generate specific amino acid chains, which later fold into three-dimensional protein structures in the cell. Ribosomes facilitate decoding by inducing the binding of complementary tRNA anticodon sequences to mRNA codons. Twenty amino acids are specified using 61 genetic codons. However, not all codons are equally used for coding amino acids. The degree of codon usage bias in viral genomes has been recognized as a key factor in regulating viral host-adaptation [8]. Classically, it is assumed that the host translational decoding machinery is static, and viruses are forced to adapt to the host through mutagenesis [9]. However, we hypothesize that the landscape of host tRNAome expression and processing is dynamic in response to viral invasion, which can be actively explored by viruses to orchestrate virus-host interactions. In this study, we investigated how human coronaviruses regulate the host mature tRNAome and the functional consequences of the infection.

## Materials and methods

### Phylogenic analysis

One representative genome from each human coronavirus species was used to construct a simplified phylogenetic tree: SARS-CoV-2 (MN908947), SARS-CoV (AY278488), MERS-CoV (KJ813439), OC43 (KX344031), 229E (KY369908), NL63 (MK334046), and HKU1 (KT779555). Sequences were aligned in MEGA 7 [10], and the tree was generated using the neighbor-joining method with 1,000 bootstrap replicates. Evolutionary distances were estimated with the p-distance model, and branch lengths were scaled accordingly. The resulting phylogenetic tree was visualized and edited using MEGA 7 [10], with bootstrap values greater than 70% shown below the branches to indicate significant support.

### Measurement of codon adaption index

Codon adaption index (CAI) of the seven coronaviruses for their, respectively, open reading frames (ORFs) 1ab, E, M, N, and S were calculated by CODONW software (http://www.molbiol.ox.ac.uk/cu, version 1.4.2) with Saccharomyces cerevisiae as reference set (matplotlib.org). These coronaviruses included 229E (Genbank: KY369908), HKU1 (Genbank: KT779555), MERS (Genbank: KJ813439), NL63 (Genbank: MK334046), OC43 (Genbank: KF530098), SARS-CoV-2 (Genbank: MN908947), and SARS-CoV (Genbank: AY278488).

### Coronavirus and human codon usage association analysis

The Relative Synonymous Codon Usage (RSCU) of the seven coronaviruses for their, respectively, open reading frames (ORFs) 1ab, E, M, N, and S were calculated by CODONW software (http://www.molbiol.ox.ac.uk/cu, version 1.4.2). RSCU values for humans were downloaded from codon usage database. The association or similarity analysis of coronavirus RSCU and human RSCU was performed by python Matplot package and Seaborn tools. Pearson correlation coefficient (*R* value) was calculated to indicate the strength of viral codon usage adaptation to their human host.

### Cell culture

Monkey LLCMK-2 cells were maintained in minimal essential medium with Earle’s salt (MEM; Gibco, Grand Island, USA) supplemented with 8% (vol/vol) heat-inactivated fetal calf serum (FCS, Sigma – Aldrich, St. Louis USA), 1% (vol/vol) nonessential amino acid (Sciencell, San Diego, California, USA), 0.1% (vol/vol) L-glutamine (Lonza, Verviers, Belgium), 100 IU/mL Penicillin, and 100 mg/mL Streptomycin (Gibco, Grand Island, USA). Human hepatoma cell line Huh7, human embryonic kidney epithelial cell line 293T and monkey kidney cell line Vero-E6 were cultured in Dulbecco’s modified Eagle medium (DMEM) (Lonza Biowhittaker, Verviers, Belgium) containing 10% (vol/vol) heat-inactivated fetal calf serum (FCS, Sigma – Aldrich, St. Louis USA), 100 IU/mL Penicillin, and 100 mg/mL Streptomycin (Gibco, Grand Island, USA). The human lung cancer cell line Calu-3 was grown in advanced DMEM/F12 supplemented with 1% (vol/vol) GlutaMAXTM Supplement (Gibco, Grandisland, USA), 10 mM HEPES (Life Technologies), 100IU/mL Penicillin and 100 mg/mL Streptomycin (Gibco, Grand Island, USA) and 10% (vol/vol) heat-inactivated fetal calf serum (FCS, Sigma – Aldrich, St. Louis USA). All cells were incubated at 37°C in a humidified atmosphere containing 5% CO_2_. Cell line authentication was performed using a short tandem repeat genotyping assay at the Molecular Diagnostics Department of Erasmus Medical Center. The mycoplasma-free status was checked regularly (commercially) and confirmed by the real-time PCR at Eurofins GATC-Biotech.

### Production of different species of infectious coronaviruses in cell culture

NL63 stock was obtained from Amsterdam UMC location AMC, University of Amsterdam, The Netherlands. OC43 and 229E strains were purchased from ATCC (USA). SARS-CoV-2 (isolate BetaCoV/Munich/BavPat1/2020; European Virus Archive Global #026 V-03883, GenBank: MT270101) was used as the wild type strain. The production and inoculation of virus were performed as previously described [11]. In brief, LLCMK-2 cells harboring infectious NL63 and Huh7 cells harboring infectious OC43 or 229E were cultured at 33°C, with 5% CO_2_ for 4–7 d. Calu-3 cells harboring infectious SARS-CoV-2 were incubated at 37°C, with 5% CO_2_ for 3–4 d. Then virus particles were harvested by repeated freezing and thawing three times and filtered with 0.45 μm filters. The culture supernatant was cleared by centrifugation and stored in aliquots at −80°C. All work with infectious SARS-CoV-2 was performed in a Class III Biosafety Lab at Erasmus Medical Center.

### Inoculation of coronaviruses

Huh7 cells were incubated with NL63 virus overnight, OC43 or 229E virus for 2 h at 33°C, SARS-CoV-2 virus for 1 h at 37°C, with 5% CO_2_. Cells were then washed with 1 × PBS (Fisher Scientific, USA) for three times to remove unattached viruses, followed by incubation in medium for different time period. Supernatant and cell lysates were collected to quantify the infectious viral titer or viral RNA, respectively.

### Quantify the total and uncharged tRNAs with a specialized qPCR method

Huh7 cells were inoculated at a multiplicity of infection (MOI) of 0.1 with different species of coronaviruses for 72 h. Total and uncharged tRNAome levels in the infected and uninfected cells were measured according to our previous publication [7]. In brief, through a deacylation reaction, the tRNAome charged amino acids are removed. The processed tRNAs were ligated with a universal DNA/RNA hybrid adaptor that can precisely ligate to the universally conserved 3’CCA terminal. After reverse transcription, then the total tRNAome can be quantified by PCR approach. Because the noncharging tRNAs have a universally free 3’CCA terminal, without coupling any amino acid on the adenine base. Their free 3’CCA terminals enable the ligation reaction between the noncharging tRNA and the tRNA adaptor that harbors a 3’ TGG overhanging. The uncharged tRNA levels were quantified by directly ligating the undecylated tRNA to the tRNA adaptor. The total and uncharged tRNAome consisting of 57 species was quantified by qRT-PCR, in which the tRNAome in the infected cells was normalized to the uninfected control, with three biological replicates (Figure S1).

### TCID50 assay

Infectious viral titers were quantified using a 50% tissue culture infectious dose (TCID50) assay, which has been described previously [11]. Briefly, 10-fold dilutions of virus were inoculated onto cells grown in 96-well tissue culture plates (Fisher Scientific, USA) at 2,000 cells/well. The plates were incubated at 33°C or 37°C for 4–7 d, and each well was examined under a light microscope for cytopathic effects (CPE). The TCID50 value was calculated using the Reed-Muench method.

### RNA isolation, cDNA synthesis, and quantitative real-time PCR (qRT-PCR)

Total RNA was extracted using Macherey-Nagel NucleoSpin® RNA II kit (Bioke, Leiden, Netherlands) and quantified using a Nanodrop ND-1000 (Wilmington, DE, USA). Complementary DNA (cDNA) was synthesized using a cDNA synthesis kit (TaKaRa Bio, Inc., Shiga, Japan). Quantitative real-time PCR was conducted using SYBR-Green-based real-time PCR (Applied Biosystems®, Austin, USA) on a StepOnePlusTM System (Thermo Fisher Scientific LifeSciences). Glyceraldehyde 3-phosphate dehydrogenase (GAPDH) gene served as the housekeeping gene. Relative expression of target gene was normalized to GAPDH using the formula 2^−ΔΔCT^, where ΔΔCT = ΔCTsample - ΔCTcontrol (ΔCT = CT [target gene] - CT[GAPDH]). Template control and reverse transcriptase control were included in all qRT-PCR experiments, and all primers were as follows, which have been used as previously described [11]: Human GAPDH sense 5'-GTCTCCTCTGACTTCAACAGCG-3'; Human GAPDH anti sense 5'-ACCACCCTGTTGCTGTAGTAGCCAA-3'; NL63 sense 5'-CTTCTGGTGACGCTAGTACAGCTTAT-3'; NL63 anti sense 5'-AGACGTCGTTGTAGATCCCTAACAT-3'; 229E sense 5’-GTCGTCAGGGTAGAATACCTTA-3'; 229E anti sense 5'-CCCGTTTGCGCTTTCTAGT-3'; OC43 sense 5'-AGCAACCAGGCTGATGTCAATACC-3′; OC43 anti sense 5'- AGCAGACCTTCCTGAGCCTTCAAT-3'; SARS-CoV-2 sense 5'-CAATGGTTTAACAGGCACAGG-3'; SARS-CoV-2 anti sense 5'-CTCAAGTGTCTGTGGATCACG-3'. For tRNA quantification, we used a previously described isolation protocol and primer sequences that we previously described (Figure S1) [7].

### Amino acid deprivation assay

Huh7 cells were cultured in DMEM without amino acids (DMEM without glutamine, without amino acids, and with 1 g/L glucose, Genaxxon Bioscience) overnight to establish an amino acid deprivation model. To further test the deprivation of each of the 20 amino acids, different amino acids except for the deprived one were added, according to the concentrations of amino acids in regular complete DMEM medium [12,13]. The culture medium containing all 20 amino acids (without deprivation) served as control. Huh7 cells were subjected to deprivation of each of the 20 amino acids in DMEM and inoculated with different species of coronaviruses. The viral RNA levels were quantified by qRT-PCR, and data were normalized to the control group without amino acid deprivation (set as 1).

### MTT assay

Huh7 cells were seeded in 96-well plates (1 × 10^4^ per well). Then treated according to experimental requirements and cultured for 48 h and 10 mM 3-(4,5-dimethylthiazol-2-yl)-2,5-diphenyltetrazolium bromide (MTT) (Sigma-Aldrich) was added. The plate was incubated at 37°C with 5% CO_2_ for 3 h, then the medium was removed, and 100 μL of DMSO (Sigma-Aldrich, St. Louis USA) was added to each well. The plate was incubated at 37°C for 30 min. The absorbance was read on the microplate absorbance reader (Bio-Rad, Hercules, CA, USA) at a wavelength of 490 nm.

### Gene knockdown by lentiviral vectors

Lentiviral pLKO knockdown vectors (Sigma-Aldrich) expressing shRNAs targeting TRNT1 or NARS1, along with appropriate controls, were obtained from the Erasmus Biomics Center and produced in HEK 293T cells. The shRNA sequences were as follows: 95#GCCTCATTATTCAAAGTACAA, 96# CCCTACTCAAATGAAGGAGAT, 97#CCCTATCAAGACTTCATTATA, 98#CGCAGAGATCTCACTATAAAT, 99#CCATTCCTCCATTTCCTGTAA; 86#CCACTCCCTATTACTGGTATA, 87# GCCTTTCAAACGGATGAACTA, 88#GCAGTGTATGGAATGCTAAAT, 89#GTGTGCTTATACCCTCGATTT, and 90#GAAGCAAAGAAGATTACCATT. The detailed method was described previously [11]. Briefly, lentiviral particles were harvested via freezing and thawing three times and filtered by 0.45 μm filters (Cytiva, USA). The harvested viruses were added to naïve Huh7 cells seeding in 12- or 6-well plates (Corning BV, Amsterdam, The Netherlands) and incubated at 37°C with 5% CO_2_ for 48 h. Stable gene knockdown cells were selected and expanded by adding puromycin (3 μg/mL) (Bio-Connect BV, Huissen, The Netherlands). TRNT1 or NARS1 RNA levels were quantified using SYBR Green – based real-time PCR assay (Thermo Fisher, USA). GAPDH was used as the housekeeping gene to normalize gene expression using the 2-ΔΔCt method. The target sequences of the selected primers were as follows: TRNT1 sense 5'-GAAGGAGATGTTTCAGTCGGCTG-3’; TRNT1 anti sense 5'-TCAGTGGTGACATCAATCCGTAG-3'; NARS1 sense 5'-AGAGCAGTCCAGAACACGAAGG-3'; and NARS1 anti sense 5'- CCACATCACAAACCAAGTCCTCC-3'.

### Codon usage and tRNA abundance association analysis

The Relative Synonymous Codon Usage (RSCU) of the four coronaviruses for their, respectively, open reading frames (ORFs) 1ab, E, M, N, and S was calculated by CODONW software (http://www.molbiol.ox.ac.uk/cu, version 1.4.2). According to the Watson–Crick base pairing rule between tRNA-anticodon and codon, RSCU values of each ORFs (1ab, E, M, N, and S) of SARS-CoV-2, 229E, OC43, and NL63 were, respectively, plotted with the levels of mature tRNAs in infected cells. The association analysis between codon usage bias and tRNAome data was analyzed and visualized by Graphpad software. Top tRNA species were filtered, in which their paired codons are frequently used (CUB > 1) and the corresponding mature tRNA levels are concurrently up-regulated.

### Statistical analysis

Statistical significance of differences between group means were evaluated using the Mann–Whitney test (GraphPad Prism; GraphPad Software Inc., La Jolla, CA, USA). The threshold for statistical significance was set at *p* < 0.05.

## Results

### The codon usage of human coronaviruses has poorly adapted to the human host

**Figure 1.**
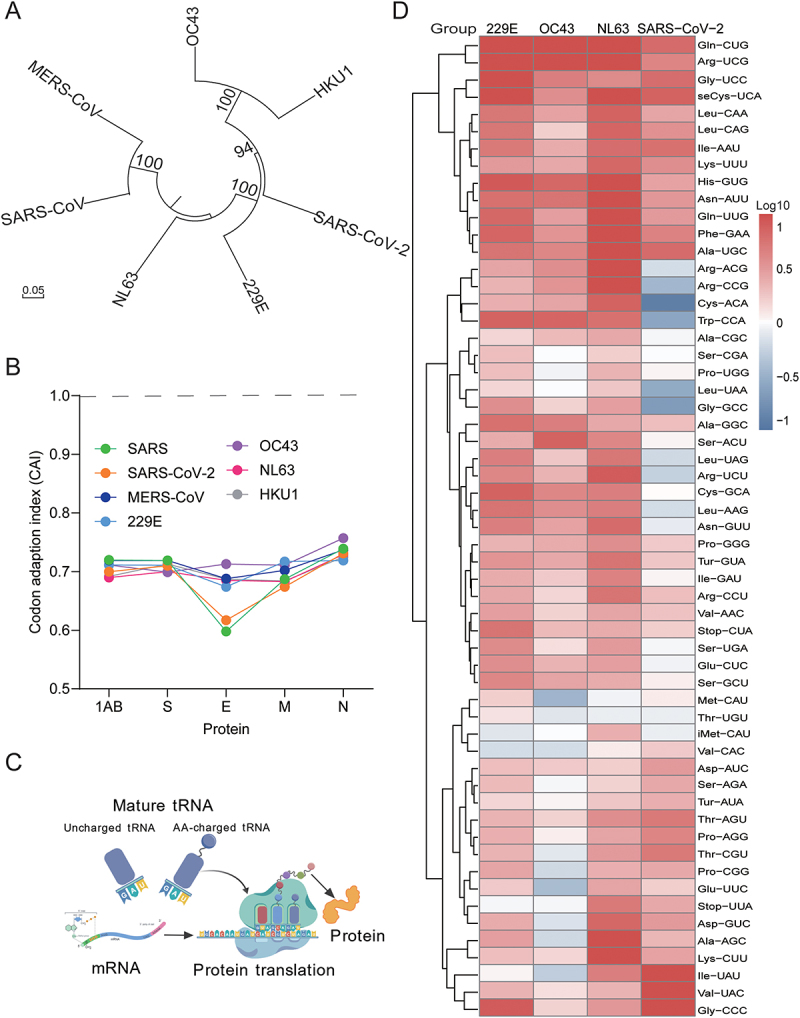
The codon usage of human coronaviruses to its human host and the landscape of the mature tRNAome upon infection. (A) Phylogenetic tree and genetic relationships of the seven species of human coronaviruses. (B) Codon adaptation index (CAI) of seven coronaviruses was analyzed for the open reading frames (ORFs) 1ab, E, M, N and S. The CAI calculation was performed by CODONW software with Saccharomyces cerevisiae as reference set. (C) Schematic illustration of mature tRNA mediated protein synthesis. The pattern was created using BioGDP.com [15]. (D) Huh7 cells were inoculated at a multiplicity of infection (MOI) of 0.1 with different species of coronaviruses for 72 h, and the mature tRNAome consisting of 57 species was quantified by qRT-PCR. Cluster analysis of the tRNAome and data were normalized to the uninfected control (CTR; set as 1) (*n* = 3).

In translational decoding, tRNA availability and codon context constitute the rate-limiting step for protein synthesis [14]. Intuitively, human coronaviruses (Figure 1(A)), although originating from animal hosts, are expected to have already adapted codon usage to fit human host codon usage patterns. Thus, we compared the relative synonymous codon usage (RSCU) of the open reading frames (ORFs) of these seven coronaviruses with that of the human host. Counterintuitively, there is no evidence that the codon usage of these coronaviruses has adapted to the human codon usage pattern for any of the analyzed ORFs, showing poor linear correlations (most of the Pearson coefficients were less than 0.20) (Figure S2).

In addition to RSCU, the codon adaptation index (CAI) has been suggested to predict the efficiency of translation elongation [16]. CAI is a measurement of the adaptiveness of the codon usage of a gene toward the codon usage of highly expressed genes, such as housekeeping genes. Our analysis indicated that the CAI of all the analyzed coronavirus ORFs remained substantially distal from the human host (Figure 1(B)). These counterintuitive results promoted us to investigate whether these coronaviruses can regulate the human tRNAome, the other side of the coin.

### A global upregulation of the host mature tRNAome landscape upon coronavirus infection

Transcribed cellular pre-tRNAs were processed, modified, and matured by the addition of CCA tails. These mature tRNAs were present in amino acid charged and uncharged forms (Figure 1(C)). We previously developed a specialized qRT-PCR method to profile a tRNAome consisting of 57 species of mature tRNAs. Through a deacylation reaction, we were able to remove the charged amino acids, and therefore the entire mature tRNAome can be quantified by this method [7,12]. Here, we inoculated human Huh7 cells with 229E, OC43, NL63, or SARS-CoV-2, which supports the replication of all these coronaviruses (Fig. S3), and harvested and quantified the total mature tRNAome 72 h post-inoculation. We found a global upregulation of the tRNAome across all infected groups, although the patterns varied among the different types of coronaviruses (Figure 1(D)). Next, we investigated the functional implications of tRNAome remodeling during coronavirus infection.

### Depriving amino acid or genetically interfering with tRNA charging inhibits coronavirus infection

**Figure 2.**
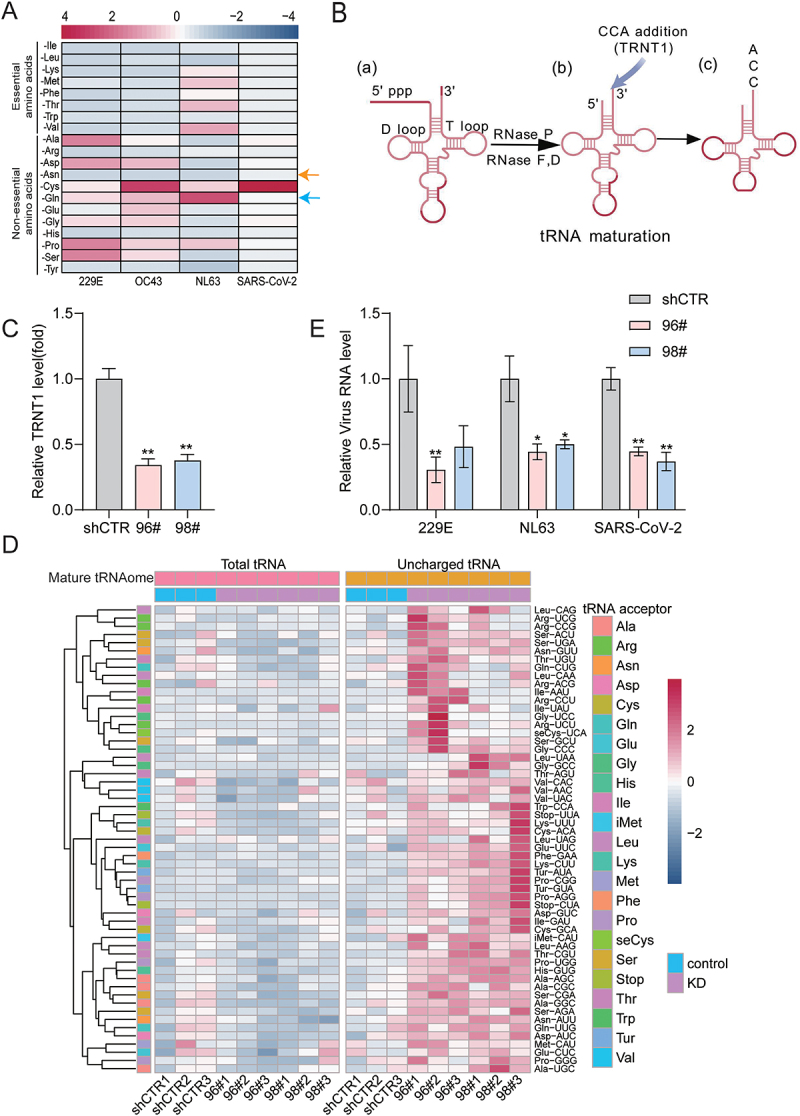
Amino acid deprivation and TRNT1 knockdown affect coronavirus infection. (A) Quantification of viral RNA levels by qRT-PCR in Huh7 cells infected with the indicated coronavirus species cultured in media deprived of the indicated amino acids for 48 h. Data were normalized to the control cultured in regular DMEM medium (*n* = 3). (B) Schematic illustration of the function of TRNT1 during tRNA maturation. The pattern was created using BioGDP.com [15]. (C) TRNT1 mRNA expression of Huh7 cells following lentiviral shRNA mediated knockdown was quantified by qRT-PCR, and the shCTR control (cells transduced with a vector without targeting any host genes) was set as 1 (*n* = 6). (D) The total and uncharged mature tRNAome consisting of 57 species was quantified by qRT-PCR of shCTR control and two TRNT1 knockdown clones (96#, 98#). Cluster analysis of the total mature tRNAome (left) and uncharged tRNAome (right) was normalized to the shCTR control (CTR; set as 1) (*n* = 3). (E) shCTR and TRNT1 knockdown cells (96#, 98#) were inoculated with the indicated coronavirus at a multiplicity of infection (MOI) of 0.1 for 48 h. The levels of viral RNA were quantified by qRT-PCR (*n* = 6–12). Data are presented as means ± SEM. *p < 0.05; **p < 0.01.

Mature tRNAs are charged with cognate amino acids for protein synthesis, and the availability of amino acids and the corresponding mature tRNAs are key factors [17]. First, we used an amino acid-free cell culture medium supplemented with the corresponding amino acid cocktail to deprive each of the 20 amino acids [12]. We observed that, in most cases, depletion of a specific amino acid resulted in inhibition of viral replication based on qRT-PCR quantification of the viral genomic RNA of 229E, OC43, NL63, and SARS-CoV-2, although the patterns varied among the different coronaviruses (Figure 2(A)). However, some peculiar cases, such as the depletion of cysteine (Cys), promoted viral replication (Figure 2(A)). Of note, amino acid deprivation has variable effects on cell viability, depending on the specific types of amino acids (Fig. S4).

tRNA-nucleotidyltransferase 1 is the only enzyme encoded by the human TRNT1 gene to add the nucleotide sequence CCA to the 3’ end of tRNA, creating a binding site for an amino acid (Figure 2(B)) [18]. We employed lentiviral vector encoding shRNA to knock down TRNT1 in Huh7 cells lines and selected two clones with significant downregulation (slightly over 50%) of TRNT1 expression (Figure 2(C)). We found that TRNT1 knockdown did not affect the abundance of total mature tRNAome, but globally increased the content of uncharged mature tRNAs, suggesting that partial loss of TRNT1 impaired tRNA maturation and amino acid charging (Figure 2(D)). Importantly, TRNT1 knockdown significantly inhibited viral replication, as shown by a significant reduction in viral RNA levels by approximately 50% in 229E, NL63, and SARS-CoV-2 (Figure 2(E)). These results demonstrate that the mature tRNAome functionally sustains the coronavirus infection.

### The up-regulated tRNA-Asn-AUU matches translational decoding need of multiple ORFs from different coronaviruses

**Figure 3.**
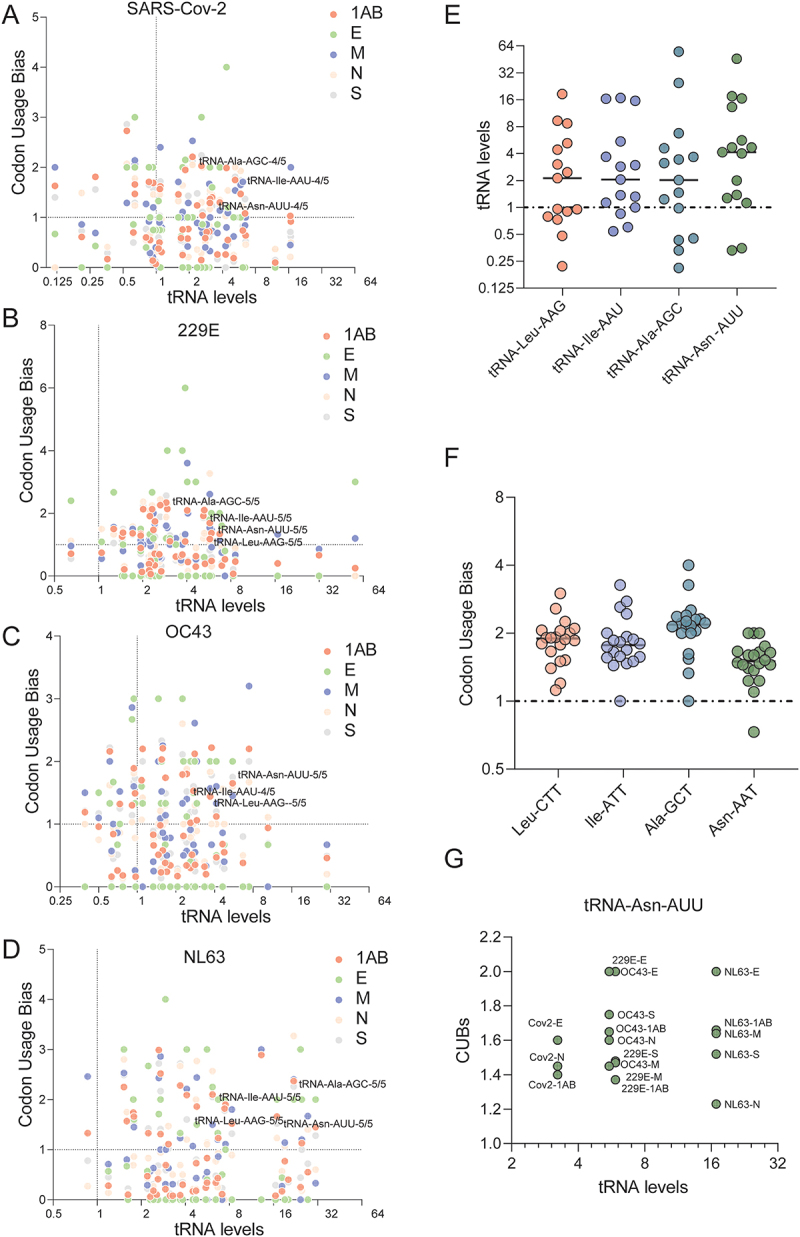
Association analysis between codon usage bias of coronaviruses and their remodeled host tRNAome landscapes. (A-D) Codon usage bias (CUBs) of SARS-CoV-2, 229E, OC43 and NL63 were plotted with the levels of mature tRnas in infected Huh7 cells. Data of codon usage bias of the open reading frames (ORFs) 1ab, E, M, N and S were labeled by different colors. (E) Top four selected tRNA species were indicated, in which their paired codons are frequently used (CUB > 1) and the corresponding mature tRNA levels are concurrently up-regulated. (F) Codon usage bias of the four coronaviruses that are associated with the top four tRnas identified in Figure 3(E) (labels for each coronavirus and their ORFs were not shown). (G) Association analysis between tRNA-Asn-AAU CUB data (Y axis) of given ORFs from different coronaviruses and the levels of mature tRNA-Asn-AUU levels (X-axis).

The mature tRNAome is bilaterally exploited by the virus and host for translational decoding. To identify the essential tRNA species that may determine coronavirus infection, we performed an association analysis between codon usage bias (CUB) of different coronaviruses and the mature tRNAome levels remodeled by the viruses, a type of need-to-supply analysis. Because viral codons with mRNAs are specifically decoded by paired tRNAs according to codon: anticodon pairing rule. As a result, we found that there are many tRNA species that can be robustly remodeled by the infection, while they complement the need for translational decoding of the four coronaviruses (CUB > 1) (Figure 3(A–D)). This finding was observed for the different ORFs (1AB, E, M, N, and S) encoded by the four coronaviruses. Among them, we found that tRNA-Leu-AAG, tRNA-Ile-AAU, tRNA-Ala-AGC, and tRNA-Asn-AUU were the top four tRNA species consistently up-regulated by the different coronaviruses (Figure 3(E)). Consistent to this analysis, the usage of the four up-regulated tRNA paired codons, among the five ORFs (1AB, E, M, N, and S) in the four coronaviruses (SARS-CoV-2, 229E, OC43, and NL63) were also above 1.0 in general, meaning that these four tRNA paired codons are favorably used in viral genome (Figure 3(F)). However, the levels of tRNA-Asn-AUU were much higher than those of others (Figure 3(E)). In addition, the up regulation of tRNA-Asn-AUU is associated with high Asn-AAT codon usage of multiple ORFs from different coronaviruses, such as the SARS-CoV-2 E gene, and 229E S gene (Figure 3(G)). This analysis indicates that tRNA-Asn-AUU may play a vital role in coronavirus infection.

### Asparagine charging functionally orchestrates coronavirus infection

**Figure 4.**
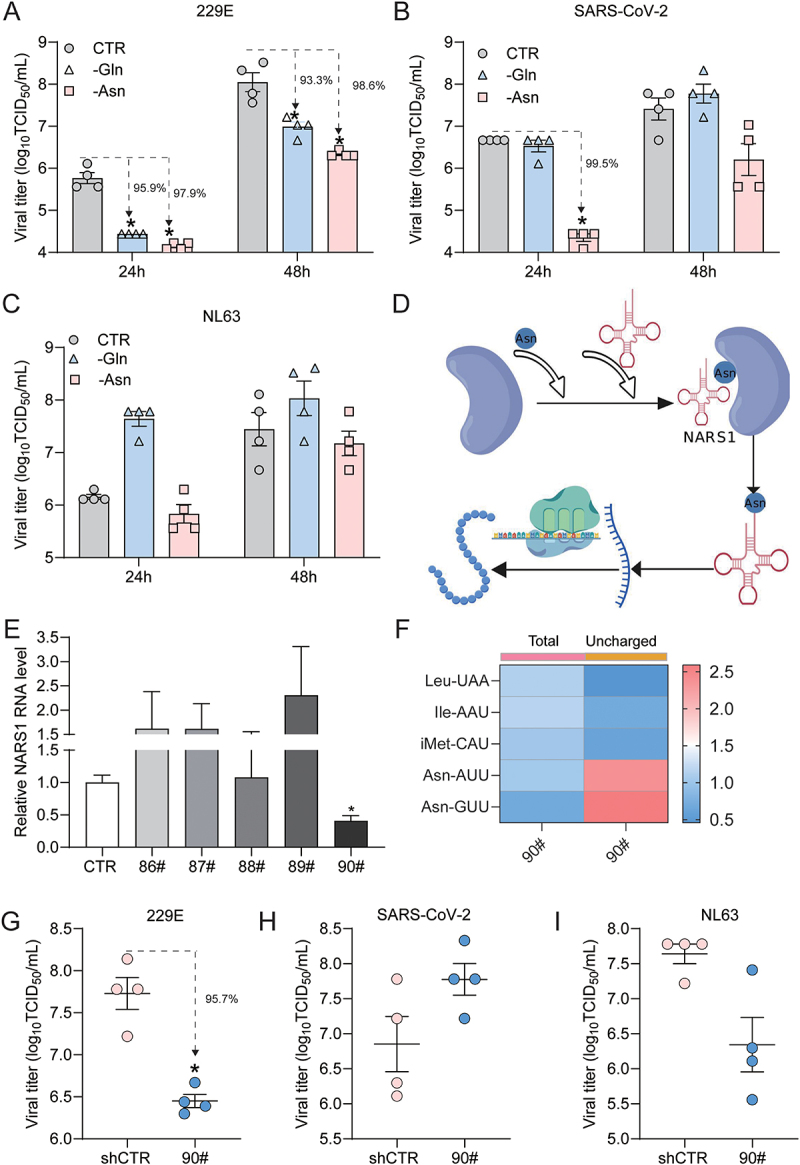
Asparagine deprivation or knockdown of asparaginyl-tRNA-synthetase affect coronavirus infection. (A-C) Huh7 cells were subjected to asparagine or glutamine deprivation, and cells cultured in regular medium served as control (CTR). Supernatant harvested 24 or 48 h post-inoculation was subjected to TCID50 assay to quantify infectious viral titers of the indicated coronaviruses (*n* = 4). (D) Schematic illustration of the process of aminoacylated tRnas biogenesis. The pattern was created using BioGDP.com [15]. (E) NARS1 mRNA expression in Huh7 cells following lentiviral shRNA mediated knockdown was quantified by qRT-PCR, and the shCTR control (cells transduced with a vector without targeting any host genes) was set as 1 (*n* = 4). (F) the selected tRnas were quantified by qRT-PCR in shCtR and Nars1 knock down cells (clone 90#). Cluster analysis of the total mature tRnas (left) and uncharged tRnas (right), and data were normalized to the shCTR control (CTR; set as 1) (*n* = 3). (G-I) shCtR and Nars1 knockdown cells (clone 90#) were inoculated with the indicated coronaviruses at a multiplicity of infection (MOI) of 0.1. Infectious viral titters were quantified by TCID50 assay 48 h post-inoculation (*n* = 4). Data are presented as means ± SEM. *p < 0.05; **p < 0.01.

Given the outstanding role of tRNA-Asn-AUU, we further validated the effects of asparagine (Asn) deprivation on infectious virus production. We compared glutamine (Gln), which plays a less prominent role (Figure 2(A) and 3). Asn deprivation profoundly inhibited the production of infectious 229E virus particles at 24 (reduction by 97.9%, *p* < 0.5) and 48 h (reduction by 98.6%, *p* < 0.5) (Figure 4(A)). Inhibition of SARS-CoV-2 was also observed at 24 h (reduction by 99.5%, *p* < 0.5), but to a lesser extent at 48 h (Figure 4(B)). The effects on NL63 were mild at both time points (Figure 4(C)). Consistently, the overall effects of Gln deprivation were milder than those of Asn (Figure 4(A–C)).

Asparaginyl-TRNA Synthetase 1 (NARS1) is an enzyme that charges the Asn amino acid onto its cognate tRNA, tRNA-Asn-AUU, and tRNA-Asn-GUU (Figure 4(D)). We employed a lentiviral vector encoding shRNA to knock down NARS1 and identified one clone with significant downregulation (approximately 50%) of NARS1 expression (Figure 4(E)). As expected, knockdown of NARS1 specifically impaired the charging of tRNA-Asn-AUU and tRNA-Asn-GUU in Huh7 cells (Figure 4(F)). This resulted in significant inhibition of infectious viral titers of 229E (reduction by 95.7%, *p* < 0.5) (Figure 4(G)), but not of SARS-CoV-2 (Figure 4(H)) or NL63 (Figure 4(I)). These results indicate that 229E is highly sensitive to the deprivation of Asn and the impairment of its charging, whereas NL63 and SARS-CoV-2 are more resilient at the level of interference with single amino acid availability or the charging process.

## Discussion

Emerging evidence has highlighted the potential role of host tRNAs in viral replication [13]. For example, specific host tRNAs are used as reverse transcription primers and are packaged into retroviral virions [19] the formation of tRNA-derived fragments upon respiratory syncytial virus infection may facilitate viral replication [20]. However, research on this topic is unique and segmented. This study revealed a novel perspective for understanding coronavirus-host interactions. Although codon usage of human coronaviruses remains poorly adapted to the human host, we found that these viruses can remodel the host tRNAome landscape to facilitate their infection and adaptation. The mature form of tRNAs performs functions in protein synthesis by charging with cognate amino acids, decoding mRNA codons, and elongating peptides. Therefore, we focused on profiling the human mature tRNAome consisting of 57 tRNA species, using a previously developed specialized qRT-PCR method [7,12]. In a human cell model, infection with 229E, OC43, NL63, or SARS-CoV-2 universally upregulates the tRNAome landscape, albeit to different degrees, with different patterns. Our assay quantified the total amount of mature tRNAs, including both charged and uncharged amino acids. Therefore, it remains unclear whether upregulation of the tRNAome landscape by coronaviruses is regulated at the level of pre-tRNA transcription or subsequent processing, requiring further research.

The availability of both individual amino acids and cognate tRNAs is a key limiting factor in translational decoding. TRNT1 is the only enzyme adding the 3’ACC tail as a scaffold for the charging of mature tRNAs with the corresponding amino acids to conduct protein synthesis [18]. As expected, by genetically knocking down TRNT1, we found increased levels of uncharged mature tRNAs, but no major effects on the abundance of the total mature tRNAome.

Given the differential patterns at the individual levels of tRNA and amino acid regulation among the tested coronaviruses, we identified a prominent role of tRNA-Asn-AUU by integrated analysis of code usage and tRNAome landscape. Deprivation of Asn or knockdown of NARS1, the enzyme charging Asn to tRNA-Asn-AUU, profoundly inhibited 229E infection, but only exerted a mild effect on NL63 or SARS-CoV-2 infection. These results suggest that NL63 and SARS-CoV-2 are likely to be more resilient to regulation at the single amino acid level. Identifying such vulnerabilities would have implications in antiviral therapeutic development, which is ideally pan-coronavirus but likely species-dependent. Fully supported by this notion, it was found that an enterocyte dependent tRNA-Ser-UGA efficiently promoted the rapid release of duck hepatitis A virus and interfered with the function of this tRNA, such as serine deprivation, indeed reduced virion release. Furthermore, we also found the depletion of Cys promote viral replication. As the precursor of glutathione (GSH), Cys is necessary for maintaining intracellular redox balance [21]. Deprivation of Cys lowers GSH levels, resulting in oxidative stress, which has been shown to facilitate viral genome replication, protein folding, and virion assembly in various systems [22]. Additionally, Cys is essential for sustaining host innate immune defenses [23]. The depletion of Cys can impair multiple redox-sensitive signaling pathways such as NF-κB and interferon responses, thereby attenuating antiviral signaling and creating a cellular environment more permissive to viral propagation [24]. In addition, amino acid deprivation therapy has been increasingly explored as a potential anti-cancer strategy [25]. Aminoacyl-tRNA synthetase targeted drug development has been explored as an antibiotic and anti-parasitic agent [26]. Our findings support the rationale for exploring the modalities as novel antiviral therapeutics.

In conclusion, we found that human coronaviruses are capable of remodeling the host mature tRNAome to facilitate infection. Unveiling the essential role of the tRNAome in orchestrating coronavirus-host interactions will open new avenues for the field to study many other pathogenic viruses in terms of cross-species transmission, host response, pathophysiology, and tRNA-targeted novel antiviral strategies.

This study has some limitations. First, our findings are based on *in vitro* models. Further validation, such as profiling the tRNAome in tissues derived from infected patients, is necessary. Second, because the TRNT1 and NARS1 enzymes are crucial for maintaining cellular physiology, complete knockout is likely lethal to the host cells. Therefore, we only achieved a limited partial knockdown, and thus the inhibitory effects on coronavirus infection likely remain under-estimated. Finally, the exact mechanisms by which these human coronaviruses remodel the tRNAome remain to be investigated.

## Supplementary Material

Supplementary_file_Clean_R1.docx

Supplementary file highlight R1.docx

## Data Availability

The datasets used and/or analyzed in the current study are available on Figshare at DOI: https://doi.org/10.6084/m9.figshare.29533607 [27].
