# Supplementary material for: Coronaviruses remodel the mature human tRNAome to modulate infection
Source: Virulence. 2025 Oct 28;16(1):2580129. doi: 10.1080/21505594.2025.2580129 (PMC12574558; doi:10.1080/21505594.2025.2580129)
Supplement: Supplementary_file_Clean_R1.docx [file KVIR_A_2580129_SM2207.docx]

**Supplementary Information for**

**Coronaviruses remodel the mature human tRNAome to modulate infection**

Yining Wang et al

Corresponding authors: q.pan@erasmusmc.nl (Q.P.); x.ou@sicau.edu.cn (X. O.)

**This file contains :**

Supplementary Figures (1-4)

**Supplementary Figure**


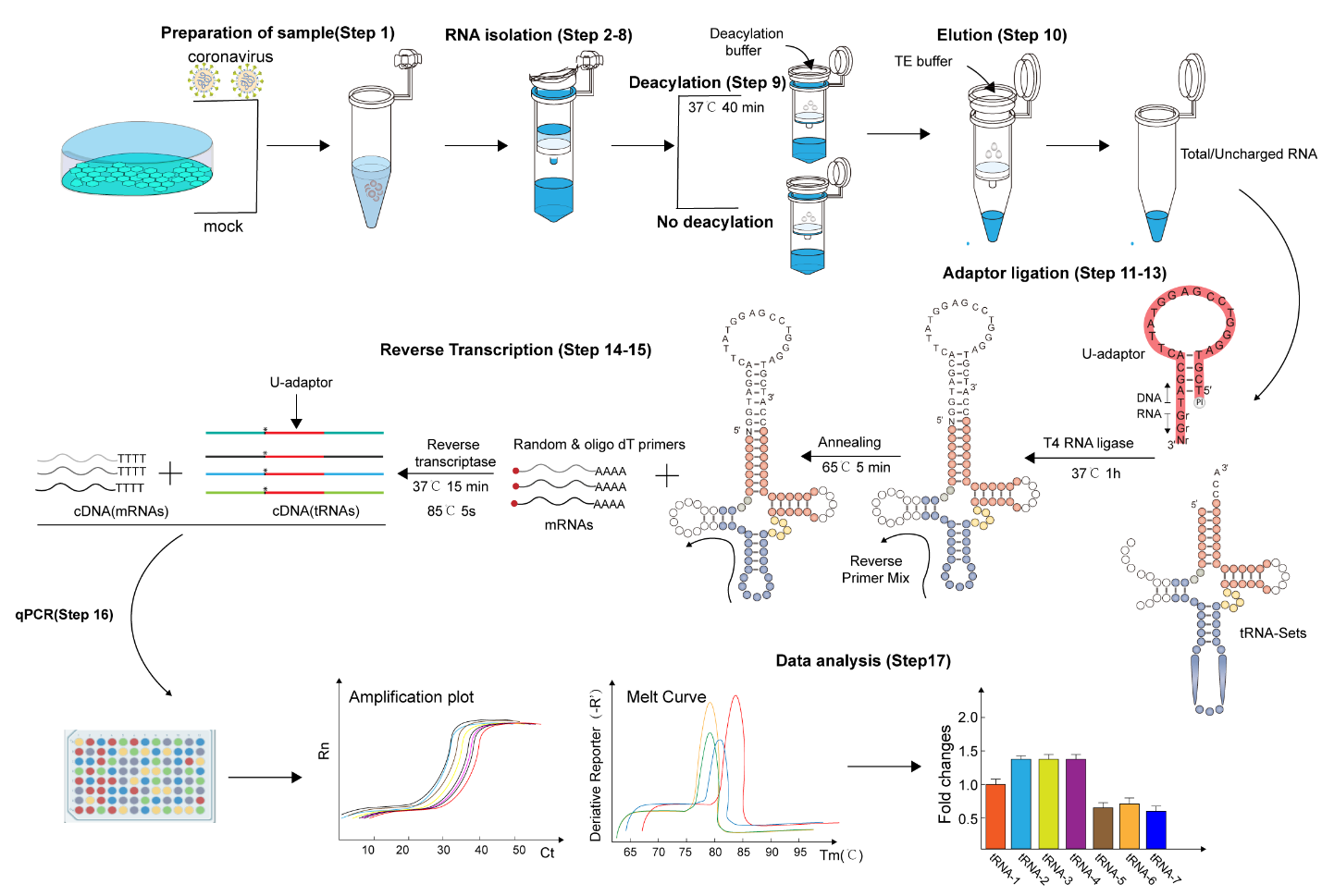


**Fig S1**. **Workflow for quantifying the tRNAome**. Total RNA was extracted from cells infected or noninfected with coronaviruses using a routine column-based RNA isolation protocol. Before elution, samples were either incubated with deacylation buffer at 37 °C for 40 minutes to remove amino acids from the tRNA CCA tail for isolating total tRNA, or not incubated with the buffer to isolate uncharged tRNA, followed by elution with TE buffer. Subsequently, tRNAs were linked to U-adaptors by T4 RNA ligase, followed by annealing with a reverse primer mix. Finally, cDNA from total mRNA and U-adaptor-linked tRNAs was synthesized concurrently for qPCR amplification. Relative tRNA expression was calculated using the 2^-ΔΔCT^ method normalized to the housekeeping gene human GAPDH.

**
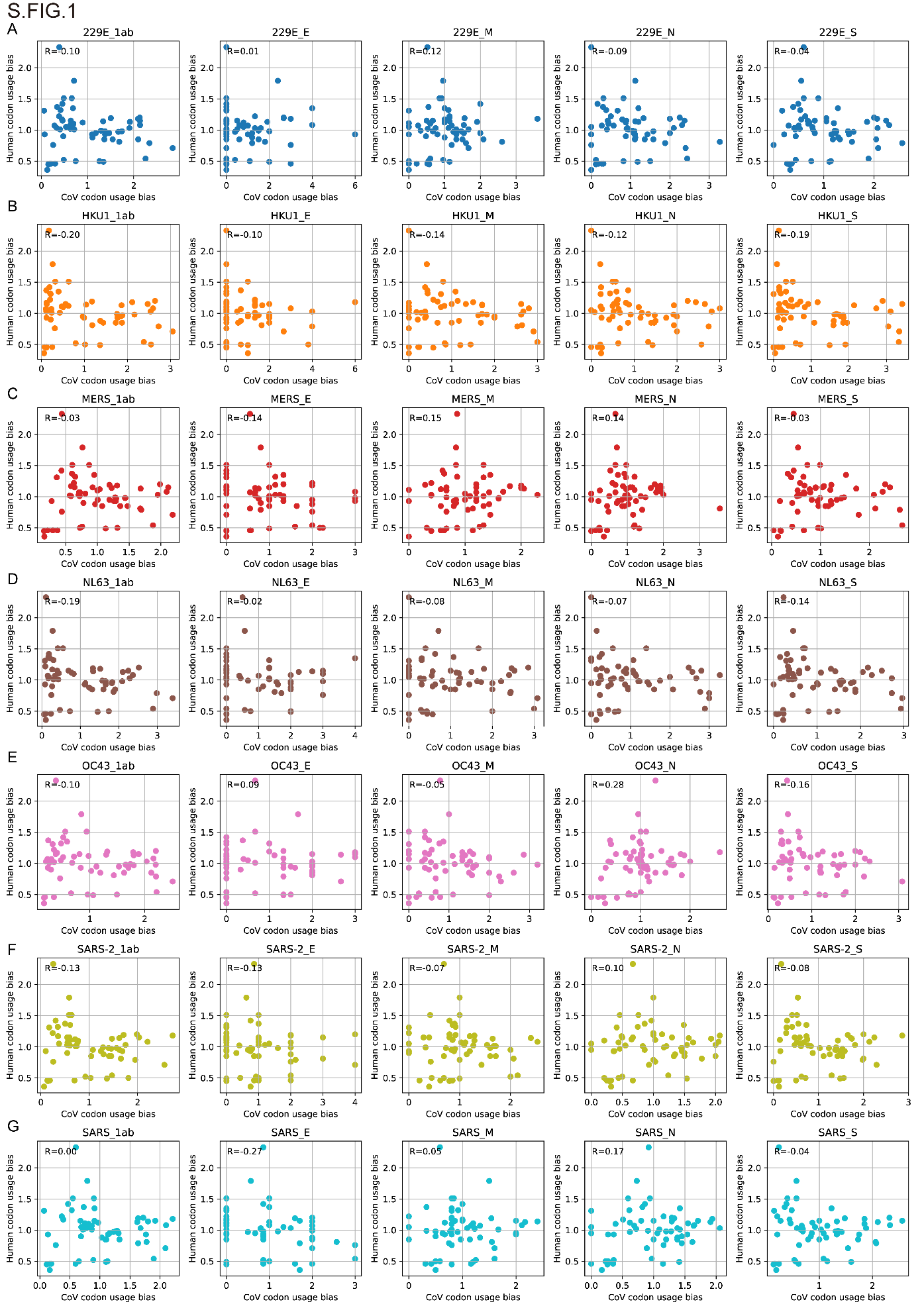
**

**Fig S2**. **The codon usage of seven human coronaviruses to its human host.** Correlation of codon usage of human coronaviruses with the overall human codon usage. For each possible codon, its relative synonymous codon usage (RSCU) in the human genome is plotted against the y-axis and the corresponding RSCU in the relevant viral open reading frames (ORFs) 1ab, E, M, N and S on the x-axis.


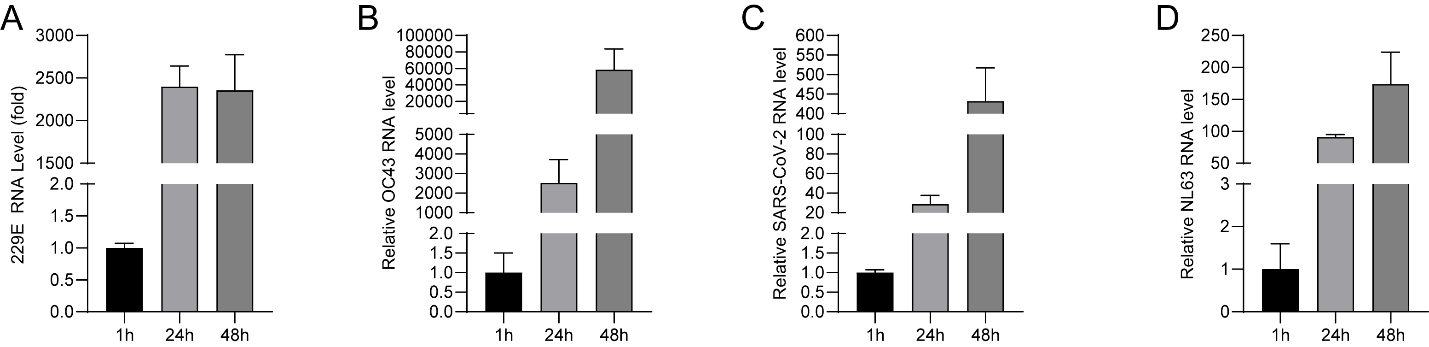


**Fig S3**. **Huh7 cells support the replication of 229E, OC43, SARS-CoV-2 and NL63 coronaviruses.** Huh7 cells were inoculated with 229E (A), OC43 (B), SARS-CoV-2 (C) and NL63 (D) for 1, 24 or 48 hours. Viral level was quantified by qRT-PCR and normalized to GAPDH reference gene (n=3, SARS-CoV-2 group, n=4, OC43, 229E and NL63 groups). Data are presented as means ± SEM.


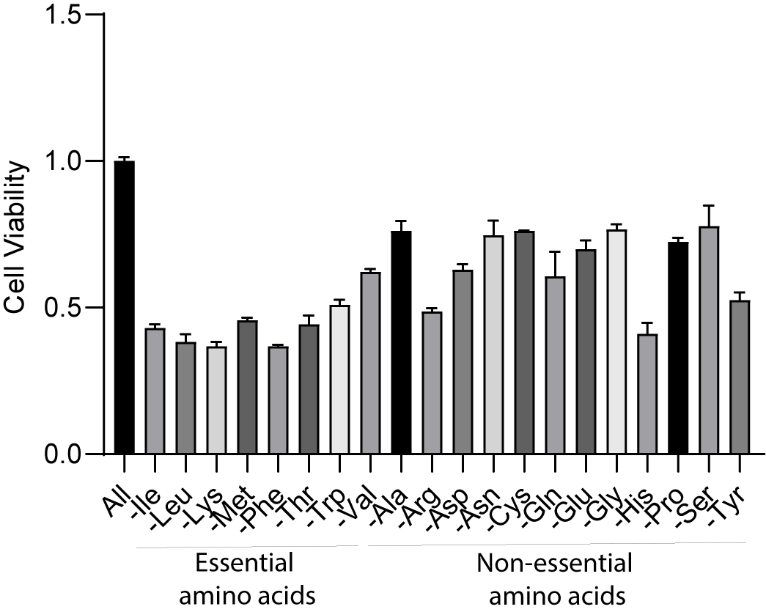


**Fig S4. The effects of amino acid deprivation on cell viability.** Huh7 cells were cultured in medium containing 20 amino acids (control) or depletion of an individual amino acid for 48 hours. MTT assay analysis of cell viability and data were normalized to the control (set as 1) (n = 4). Data are presented as means ± SEM.
